# *Iris germanica* L. Rhizome-Derived Exosomes Ameliorated Dihydrotestosterone-Damaged Human Follicle Dermal Papilla Cells Through the Activation of Wnt/β-Catenin Pathway

**DOI:** 10.3390/ijms26094070

**Published:** 2025-04-25

**Authors:** Mujun Kim, Jung Woo, Jinsick Kim, Minah Choi, Hee Jung Shin, Youngseok Kim, Junoh Kim, Dong Wook Shin

**Affiliations:** 1 Research Institute for Biomedical and Health Science, Konkuk University, Chungju 27478, Republic of Korea; besy100@kku.ac.kr (M.K.); jindoli477@kku.ac.kr (J.K.); 2 Shinsegae International Inc., Seoul 06015, Republic of Korea; friendship94@sikorea.co.kr (J.W.); minah0913@sikorea.co.kr (M.C.); hee-jungshin@sikorea.co.kr (H.J.S.); yskim23@sikorea.co.kr (Y.K.); junohkim@sikorea.co.kr (J.K.)

**Keywords:** anti-hair loss, dihydrotestosterone, exosome, human follicle dermal papilla cells, *Iris germanica* L. rhizome, Wnt/β-catenin pathway

## Abstract

Hair loss is often associated with oxidative stress and mitochondrial dysfunction in human follicle dermal papilla cells (HFDPCs), resulting in impaired cellular function and follicle degeneration. Thus, many studies have been conducted on natural plants aimed at inhibiting hair loss. This study investigated the therapeutic potential of exosomes derived from the rhizomes of *Iris germanica* L. (Iris-exosomes) in HFDPCs damaged by dihydrotestosterone (DHT). Iris-exosomes significantly reduced reactive oxygen species (ROS) levels, restoring mitochondrial membrane potential and ATP production, thereby mitigating oxidative stress and improving mitochondrial function. These effects occurred alongside enhanced cellular processes critical for hair follicle regeneration, including increased cell migration, alkaline phosphatase (ALP) activity, and three-dimensional (3D) spheroid formation, which replicates the follicle-like microenvironment and promotes inductive potential. Furthermore, Iris-exosomes stimulated the Wnt/β-catenin signaling pathway by enhancing glycogen synthase kinase-3β (GSK-3β), AKT, and extracellular signal-regulated kinase (ERK), leading to β-catenin stabilization and nuclear translocation, thereby supporting the expression of genes essential for hair growth. Taken together, these findings suggest that Iris-exosomes can be promising ingredients for alleviating hair loss.

## 1. Introduction

Exosomes are nanoscale extracellular vesicles (30–400 nm) released by most animal and plant cell types via the inward budding of the endosomal membrane during the formation of multivesicular bodies (MVBs) [1,2]. These MVBs merge with the plasma membrane, enabling the release of exosomes into the extracellular space [3]. Exosomes play a crucial role in intercellular communication, mediating the transfer of bioactive molecules such as proteins, lipids, mRNAs, and microRNAs between cells [4,5]. Exosomes possess a unique ability to carry molecular signals and influence the behavior of recipient cells, which sustain their roles in both physiological and pathological processes, including immune modulation and tissue regeneration [6,7]. Exosomes have emerged as potential therapeutic agents in regenerative medicine [8,9]. Notably, mesenchymal stem cell (MSC)-derived exosomes have demonstrated promising effects in enhancing cell proliferation, migration, and tissue repair, highlighting their application in regenerative therapies [10,11]. Similarly, exosomes from dermal papilla cells have shown efficacy in promoting hair growth by enhancing the activity of follicular keratinocytes and increasing the secretion of key growth factors [12]. Recent studies also suggest that plant-derived exosomes, containing functional biomolecules such as mRNAs and secondary metabolites, hold potential as biocompatible and scalable therapeutic agents, particularly in anti-inflammatory and antioxidative applications [13,14]. These findings underscore the significant potential of exosomes as bioactive agents in cosmetic science, presenting innovative approaches to enhance skin and hair health [15].

Androgenetic alopecia (AGA) represents the most prevalent type of hair loss [16,17]. It is characterized by a progressive thinning of hair in androgen-sensitive regions, including the frontal, temporal, and vertex areas of the scalp, resulting from the miniaturization of hair follicles [18,19]. This condition arises from the interaction between genetic predisposition and androgen hormones, particularly DHT [20,21]. As a derivative of testosterone, DHT binds to androgen receptors in susceptible hair follicles, initiating a cascade of events that shortens the anagen phase of the hair cycle and promotes follicular miniaturization [22,23]. The pathophysiology of AGA involves the upregulation of 5-alpha-reductase, the enzyme responsible for converting testosterone to DHT in the hair follicle [24]. Current treatment options, including 5-alpha-reductase inhibitors like finasteride and topical vasodilators like minoxidil (MIX), target these pathways but often have limited efficacy and potential side effects, underscoring the need for novel therapeutic strategies [25,26,27].

HFDPCs, mesenchymal cells with specialized functions positioned at the base of hair follicles, play a pivotal role in hair growth and follicular morphogenesis [28,29]. Due to their ability to secrete growth factors that influence the hair cycle, HFDPCs have become indispensable in models for studying hair loss and evaluating hair growth-promoting agents [30,31]. These models not only provide a controlled environment for understanding hair follicle biology but also serve as an effective platform for screening potential therapeutic agents targeting hair follicle regeneration [32,33].

The Wnt/β-catenin pathway plays a crucial role in hair follicle stem cell activation and dermal papilla cell proliferation, which are essential for initiating and maintaining the anagen phase of the hair cycle [34,35]. Recent studies indicate that DHT, the primary androgen involved in AGA, inhibits Wnt/β-catenin signaling, leading to follicular miniaturization and progressive hair loss [36,37]. Given this mechanism, restoring Wnt/β-catenin activity has emerged as a promising therapeutic strategy for AGA, as it may promote follicular regeneration, sustain dermal papilla cell proliferation, and prolong the anagen phase, ultimately enhancing hair regrowth [38,39].

Recent studies have demonstrated that plant-based compounds may help alleviate hair loss by targeting key pathways involved in hair follicle regeneration. Botanical extracts such as saw palmetto and rosemary extracts exhibit anti-androgenic, antioxidant, and anti-inflammatory properties, supporting hair growth and follicular health [40,41]. Plant-derived bioactive compounds, with their multimodal mechanisms and potentially favorable safety profiles, may serve as alternative therapies for hair loss management [42].

*Iris* species, particularly *Iris germanica* L., have been extensively studied for their diverse pharmacological properties, including antioxidant, anti-inflammatory, antimicrobial, and neuroprotective effects [43,44,45]. These benefits are attributed to various bioactive compounds, such as flavonoids, phenolics, and isoflavones, found in their rhizomes, flowers, and leaves [46,47]. Although the pharmacological properties of *Iris* extracts are well documented, their potential effects on hair growth remain limited.

Thus, we investigated whether exosomes derived from *Iris germanica* L. rhizomes could improve DHT-damaged HFDPCs, aiming to evaluate their potential as a therapeutic agent for mitigating hair loss.

## 2. Results

### 2.1. Characterization of Iris Exosomes Derived from Iris germanica L. Rhizome

**Figure 1:**
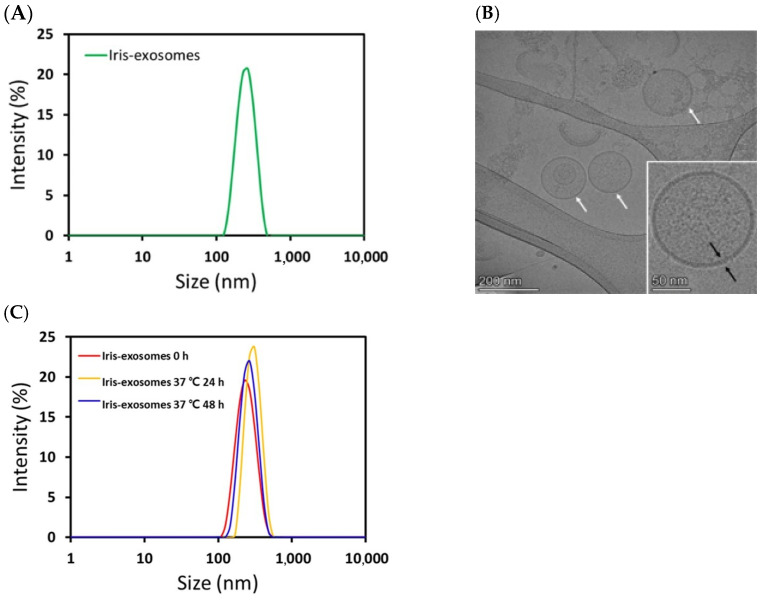
Characterization of Iris-exosomes isolated from *Iris germanica* L. rhizome. (**A**) Size distribution of Iris-exosomes. (**B**) Representative cryo-TEM images of Iris-exosomes at different magnifications. White arrow: exosomes, black arrow: lipid double layer (scale bar 200 nm and 50 nm, respectively). (**C**) The size of Iris-exosomes was measured after storage at 37 °C for 0, 24, and 48 h. These data are representative of three independent experiments.

An aqueous two-phase system with polyethylene glycol (PEG) and dextran was employed to isolate Iris-exosomes from the *Iris germanica* L. rhizome. Before evaluating their efficacy, physical characterization of the obtained Iris-exosomes was performed using dynamic light scattering (DLS), cryogenic transmission electron microscopy (cryo-TEM), and nanoparticle tracking analysis (NTA). The DLS and cryo-TEM results revealed that Iris-exosomes had an average hydrodynamic particle size of 261.7 ± 6.409 nm (Figure 1A), with a uniform and round morphology (Figure 1B). The DLS results further showed that the particle size was 259.7 ± 3.34 nm (polydispersity index (PDI), 0.3223 ± 0.02681) at 0 h. After storage, the measured particle size was 286.8 ± 3.34 nm (PDI, 0.3282 ± 0.01606) at 24 h and 277.6 ± 9.607 nm (PDI, 0.2804 ± 0.01016) at 48 h (Figure 1C), indicating that there was no significant change in particle size over time. According to NTA measurement, the concentration of Iris-exosomes was confirmed to be 1.69 × 10^9^ particles/mL, further validating their successful isolation and characterization.

### 2.2. Effect of Iris-Exosome Treatment on the Cell Viability of HFDPCs

**Figure 2:**
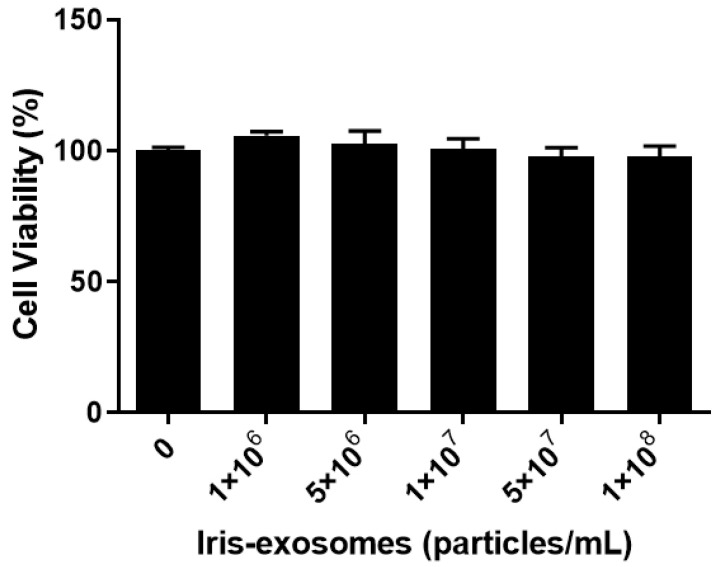
The cell viability of HFDPCs treated with Iris-exosomes. Cell viability was assessed via the MTT assay and calculated as a percentage (%) relative to the untreated control group. The data are presented as mean ± SD (*n* = 3).

To assess the cytotoxicity of Iris-exosomes on HFDPCs, MTT assays were conducted at various concentrations for 24 h. The cell viability assay was used to determine the appropriate concentration of Iris-exosomes for subsequent experiments. Iris-exosomes were not cytotoxic to HFDPCs at the tested concentrations, ranging from 10^6^ particles/mL to 10^8^ particles/mL (Figure 2).

### 2.3. Iris-Exosomes Increase the Migration of DHT-Damaged HFDPCs

**Figure 3:**
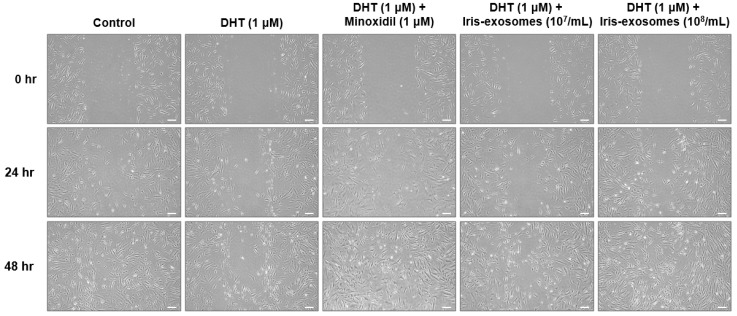
The wound healing effect of Iris-exosomes on HFDPCs stimulated by 1 µM DHT. Cells were treated with Iris-exosomes (10^7^ and 10^8^ particles/mL) or 1 µM MIX for 48 h, respectively. Wound closure was observed using a phase-contrast microscope at 24 h and 48 h, respectively, and the representative images from three independent experiments are shown (scale bar 20 µm).

The activity of dermal papilla cells, including their proliferation and migration, is critical for hair follicle regeneration and growth [48]. These processes are considered key factors in maintaining and extending the anagen phase of the hair cycle [49]. The effect of Iris-exosomes on the migration of DHT-damaged HFDPCs was evaluated using a wound healing assay. The DHT-treated group exhibited slower wound closure compared to the control, indicating that DHT impaired cell migration. In contrast, the wound distance decreased after 48 h of treatment with Iris-exosomes and MIX, compared with the DHT-treated group, meaning that Iris-exosomes enhanced wound closure. Consequently, we demonstrated that Iris-exosomes increased cell migration in a concentration-dependent manner (Figure 3).

### 2.4. Iris-Exosomes Enhance Alkaline Phosphatase Expression in DHT-Damaged HFDPCs

**Figure 4:**
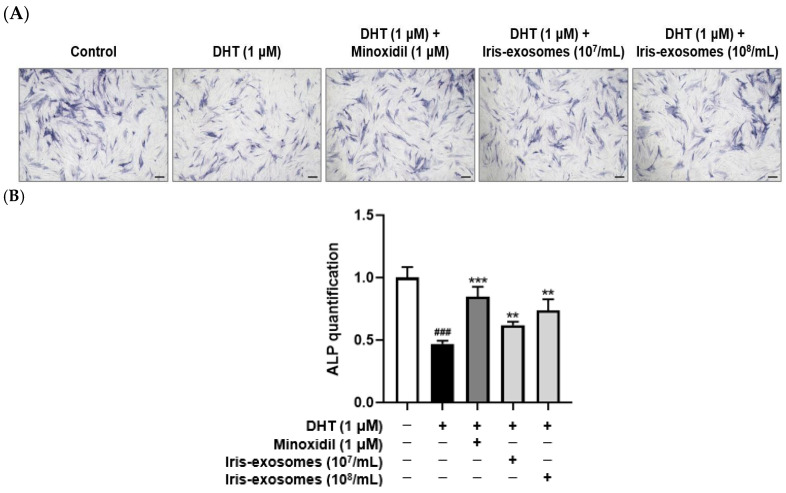
Effects of Iris-exosomes on alkaline phosphatase expression levels in HFDPCs stimulated by 1 µM DHT. Cells were treated with Iris-exosomes (10^7^ and 10^8^ particles/mL) or 1 µM MIX for 24 h. (**A**) Representative alkaline phosphatase staining images, showing results from one of three independent experiments (scale bar 20 µm). (**B**) The expression levels of alkaline phosphatase were quantified using ImageJ software, version 1.53e. Results are presented as mean ± SD (*n* = 3), with statistical significance denoted as ** *p* < 0.01 and *** *p* < 0.001 relative to the DHT-treated group. ### *p* < 0.001 compared with the control group.

Alkaline phosphatase is highly expressed in HFDPCs, with its activity peaking during the early anagen phase [50]. Since alkaline phosphatase activity promotes the hair follicle cycle, it is regarded as an indicator of hair inductivity [51,52]. The DHT-treated group exhibited lower alkaline phosphatase expression than the untreated control, suggesting that DHT impairs key markers associated with the hair cycle. In contrast, previous studies have shown that MIX increases alkaline phosphatase expression, thereby promoting the hair cycle [53,54]. As expected, treatment with 1 µM MIX elevated alkaline phosphatase expression levels in DHT-damaged HFDPCs. Similarly, Iris-exosomes elevated alkaline phosphatase levels compared with the group treated with DHT alone (Figure 4).

### 2.5. Iris-Exosomes Reduce ROS Levels in DHT-Damaged HFDPCs

**Figure 5:**
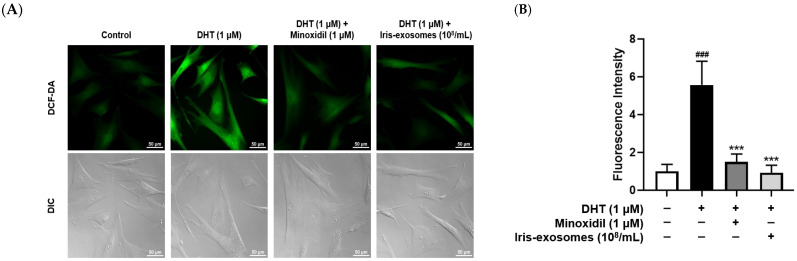

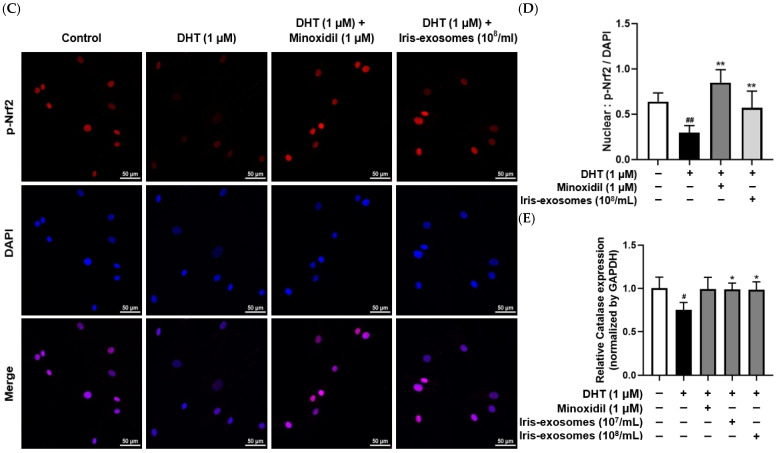
Effects of Iris-exosomes on ROS levels in HFDPCs stimulated by 1 µM DHT. (**A**,**B**) Representative fluorescence images from DCF-DA staining are presented, accompanied by quantification of fluorescence intensity. Green fluorescence indicates intracellular ROS levels (scale bar 50 µm). (**C**,**D**) The expression level of p-Nrf2 was assessed through immunofluorescence analysis. The nuclear p-Nrf2 (red) was quantified as the ratio of p-Nrf2 to DAPI (blue) (scale bar 50 µm). (**E**) The expression of catalase mRNA was assessed (*n* = 3). Data are presented as mean ± SD (*n* = 3), with statistical significance denoted by * *p* < 0.05, ** *p* < 0.01, and *** *p* < 0.001 relative to the DHT-treated group. # *p* < 0.05, ## *p* < 0.01, and ### *p* < 0.001 compared with the control group.

Skin aging results from both intrinsic and extrinsic environmental factors [55]. In particular, extrinsic aging is driven by ROS generated from environmental sources, such as UV radiation, stress, and pollution [56]. Although ROS plays a role in normal redox regulation, excessive accumulation can lead to cellular damage and contribute to various diseases [57]. Exposure of HFDPCs to ROS disrupts hair growth and maintenance processes, leading to hair loss [58]. Therefore, to assess the effect of Iris-exosomes on ROS levels in HFDPCs, we performed a DCF-DA assay. As expected, ROS levels were increased in DHT-damaged HFDPCs compared with the control group. In contrast, MIX treatment decreased ROS levels in the DHT-treated group. We also observed that Iris-exosomes significantly decreased DHT-induced ROS levels in HFDPCs, restoring them to levels similar to the control group (Figure 5A,B).

Nrf2 plays a crucial role in the cellular defense against oxidative stress by regulating the transcription of various antioxidant enzymes, including catalase [59,60]. Translocation of phosphorylated Nrf2 (p-Nrf2) to the nucleus was evaluated by immunofluorescence staining. We demonstrated that Iris-exosomes enhanced the activity of Nrf2 and the expression of catalase, which were downregulated in response to DHT treatment (Figure 5C–E).

### 2.6. Iris-Exosomes Restore the Mitochondrial Membrane Potential in DHT-Damaged HFDPCs

**Figure 6:**
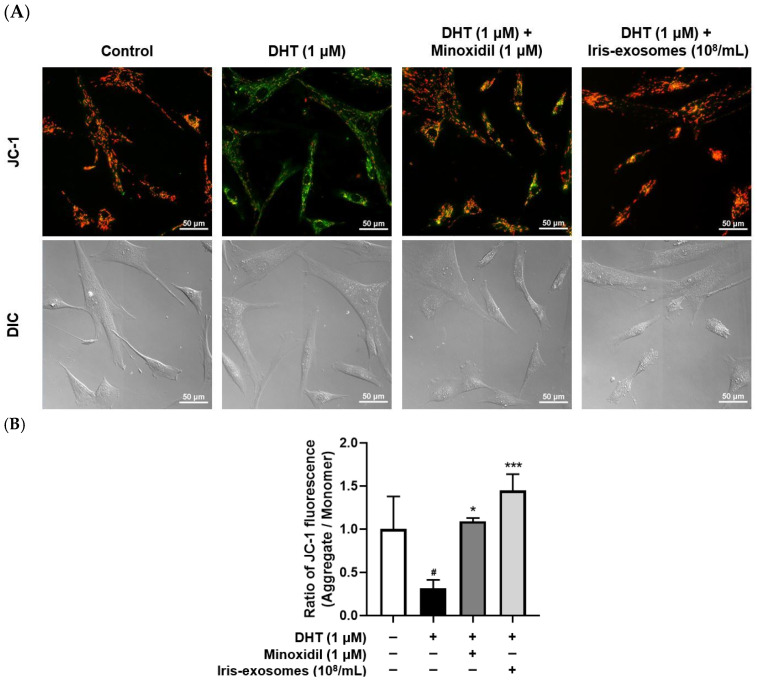
Effects of Iris-exosomes on mitochondrial membrane potential in HFDPCs stimulated by 1 µM DHT. The JC-1 assay was conducted on HFDPCs treated with 1 µM DHT, followed by treatment with Iris-exosomes (10^8^ particles/mL) or 1 µM MIX for 24 h. (**A**) Representative JC-1 fluorescence images. Green fluorescence represents depolarized mitochondria, while red fluorescence indicates hyperpolarized mitochondria. Images represent data from three independent experiments (scale bar 50 µm). (**B**) Mitochondrial membrane potential was quantified using ImageJ software, version 1.53e. Data are presented as mean ± SD (*n* = 3), with statistical significance denoted as * *p* < 0.05 and *** *p* < 0.001 relative to the DHT-treated group. # *p* < 0.05 compared with the control group.

Mitochondria are essential for energy generation, and mitochondrial dysfunction is associated with the development of various diseases [61]. The anagen phase requires significant energy and metabolic support [62]. However, mitochondrial dysfunction can disrupt the hair cycle, potentially causing hair loss [63]. We performed a JC-1 assay to evaluate the mitochondrial membrane potential in DHT-damaged HFDPCs following Iris-exosome treatment. Green fluorescence indicates impaired membrane potential, while red fluorescence represents healthy mitochondrial membrane potential. As expected, DHT treatment resulted in higher green fluorescence levels compared with the control group. However, Iris-exosomes treatment enhanced red fluorescence in DHT-damaged HFDPCs, suggesting that Iris-exosomes restored the mitochondrial membrane potential (Figure 6).

### 2.7. Iris-Exosomes Enhance ATP Levels in DHT-Damaged HFDPCs

**Figure 7:**
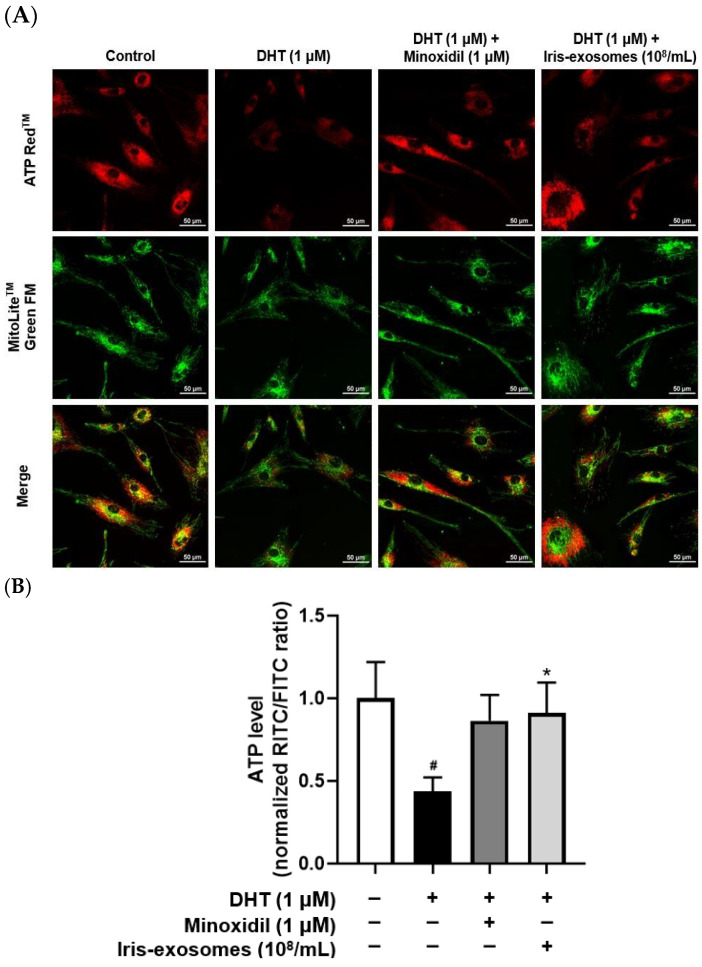
Effects of Iris-exosomes on ATP levels in HFDPCs stimulated by DHT. The ATP assay was conducted on HFDPCs treated with 1 µM DHT, followed by treatment with Iris-exosomes (10^8^ particles/mL) or 1 µM MIX for 24 h. (**A**) Representative fluorescence images. Red fluorescence represents ATP levels, while green fluorescence indicates mitochondria. Images represent one of three independent experiments (scale bar 50 µm). (**B**) ATP levels were analyzed using ImageJ software, version 1.53e, and data are presented as mean ± SD (*n* = 3). Statistical significance is marked as * *p* < 0.05 relative to the DHT-treated group. # *p* < 0.05 compared with the control group.

ATP serves as the primary energy source for cellular activities, including cell division, migration, and the secretion of signaling molecules [64]. In dermal papilla cells, reduced ATP levels impair these essential processes, ultimately weakening hair growth and potentially leading to hair loss [65]. Furthermore, mitochondrial dysfunction in dermal papilla cells worsens this issue by reducing ATP production and impairing hair follicle regeneration [62,66,67]. We assessed ATP activity in DHT-damaged HFDPCs treated with Iris-exosomes using fluorescence microscopy. Red and green fluorescence represent ATP and mitochondria, respectively. Red fluorescence intensity was reduced in DHT-damaged HFDPCs compared with the control group. However, Iris-exosomes treatment increased the intensity of red fluorescence, indicating the restoration of ATP levels (Figure 7).

### 2.8. Iris-Exosomes Upregulate the Phosphorylation Levels of AKT, ERK, and GSK-3β and the Expression of β-Catenin in DHT-Damaged HFDPCs

**Figure 8:**
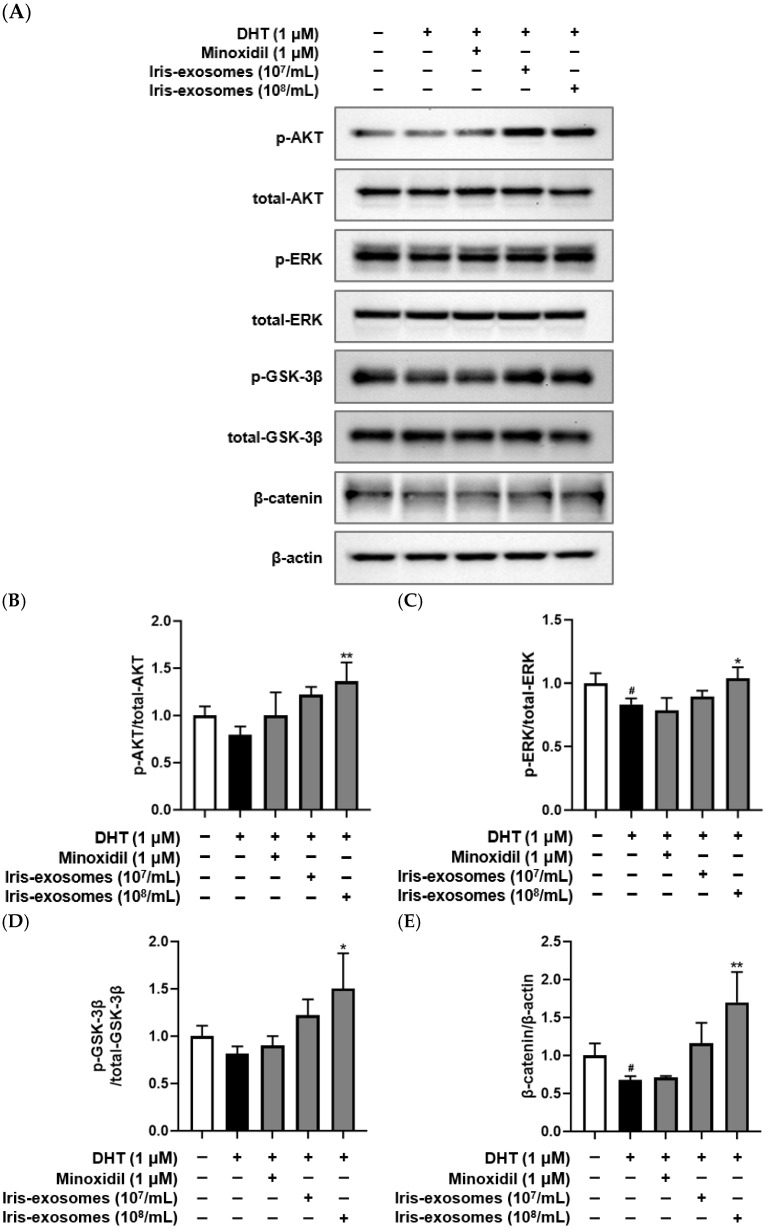
Effects of Iris-exosomes on the phosphorylation levels of AKT, ERK, GSK-3β, and the expression of β-catenin in DHT-damaged HFDPCs. (**A**) Representative Western blot images showing the relative expression levels of each protein. (**B**) AKT, (**C**) ERK, (**D**) GSK-3β, and (**E**) β-catenin relative expression bar graphs. HFDPCs were treated with 1 µM DHT, followed by Iris-exosomes (10^7^ and 10^8^ particles/mL) or 1 µM MIX for 24 h. Protein levels were analyzed using Western blotting. The results are expressed as mean ± SD (*n* = 3). Statistical significance is indicated by * *p* < 0.05 and ** *p* < 0.01 relative to the DHT-treated group. # *p* < 0.05 compared with the control group.

The Wnt/β-catenin signaling pathway is critical for regulating hair follicle development and regeneration [68]. Wnt proteins interact with a low-density lipoprotein-related protein and a frizzled receptor or receptor tyrosine kinase [69]. The receptor tyrosine kinase enhanced GSK-3β activity by phosphorylating the upstream kinases ERK or AKT, prompting the translocation of β-catenin into the nucleus [70,71]. This translocation subsequently induced the expression of genes involved in hair proliferation [72]. We performed a Western blot assay to assess the effects of Iris-exosomes on the activation of AKT, ERK, and GSK-3β through phosphorylation and the upregulation of β-catenin expression. The phosphorylation levels of AKT, ERK, and GSK-3β, along with the expression of β-catenin, were reduced in DHT-damaged HFDPCs compared with the control group. On the contrary, treatment of Iris-exosomes increased the phosphorylation of AKT, ERK, and GSK-3β and the upregulation of β-catenin expression compared with the DHT-treated group (Figure 8). These findings suggest that Iris-exosomes alleviate hair loss through the activation of AKT/ERK and Wnt signaling pathways in DHT-damaged HFDPCs.

### 2.9. Iris-Exosomes Increase the 3D Spheroid Size in DHT-Damaged HFDPCs

**Figure 9:**
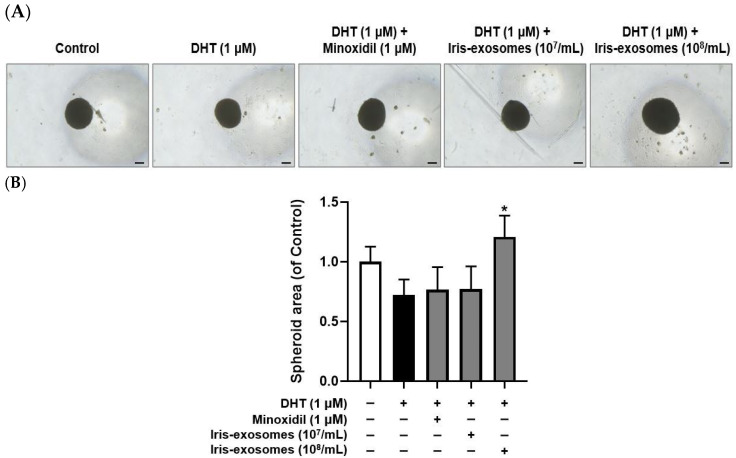
Effects of Iris-exosomes on the formation of 3D spheroid in DHT-damaged HFDPCs. Treatments with 1 µM DHT, 1 µM MIX, and Iris-exosomes (10^7^ and 10^8^ particles/mL) were administered at 2-day intervals. Images were captured after 21 days of culture. (**A**) Representative 3D spheroid images were acquired using a phase-contrast microscope, showing results from one of three independent experiments (scale bar 20 µm). (**B**) Quantification of 3D spheroid size was conducted using ImageJ software, version 1.53e. Data are presented as mean ± SD (*n* = 3), with statistical significance marked as * *p* < 0.05 compared with the DHT-treated group.

An alternative in vitro approach to enhancing the trichogenic potential of HFDPCs is the development of 3D spheroid cultures [73]. These cultures restore cell–cell interactions and enhance the in vivo hair-inductive capacity of HFDPCs [28,74]. Compared with two-dimensional cultures, this model enables a deeper understanding of complex cellular behaviors [75]. As expected, spheroid size was decreased in the DHT-treated group compared with the control group. However, treatment with Iris-exosomes increased spheroid size relative to DHT-damaged HFDPCs, meaning the beneficial effects of Iris-exosomes on HFDPCs (Figure 9).

## 3. Discussion

Hair growth studies rely on HFDPCs due to their pivotal role in regulating the hair cycle and driving follicular morphogenesis [76,77]. Markers such as alkaline phosphatase and cell migration activity are commonly used to assess the functional state of HFDPCs [78,79]. Alkaline phosphatase expression correlates with hair follicle inductivity, particularly during the early anagen phase [80]. Similarly, the migratory capacity of HFDPCs plays a significant role in their interaction with surrounding epithelial cells, influencing hair follicle formation [81]. Furthermore, their ability to aggregate into 3D spheroids is crucial for maintaining their inductive potential, as spheroid formation mimics the natural microenvironment of dermal papilla cells and enhances their trichogenic properties [82]. Thus, we investigated that Iris-exosomes enhanced hair growth in DHT-damaged HFDPCs by promoting key cellular functions and supporting follicle-like structures. Treatment with Iris-exosomes significantly stimulated cell migration in DHT-damaged HFDPCs, an essential process for hair follicle regeneration (Figure 3). Furthermore, Iris-exosomes elevated the activity of alkaline phosphatase, a critical biomarker for hair follicle inductivity (Figure 4). Additionally, we observed that Iris-exosomes significantly enhanced 3D spheroid formation in DHT-damaged HFDPCs, a model that effectively replicates cell–cell interactions and signaling pathways within dermal papilla cells. The enlargement of spheroid size observed with Iris-exosome treatment indicated their ability to promote hair growth by reinforcing dermal papilla cell activity in a tissue-mimicking environment (Figure 9).

ROS and mitochondrial dysfunction significantly influence hair growth and are implicated in various forms of hair loss. When ROS is generated in excess, it can cause cellular damage, thereby impairing the function of hair follicle cells [83]. Additionally, mitochondria play a pivotal role in providing energy for cellular processes, particularly during the anagen phase of the hair cycle, as this stage requires high levels of ATP to support rapid cell proliferation and metabolic activity [84]. However, mitochondrial dysfunction not only leads to impaired ATP production but also results in excessive ROS generation, which disrupts cellular signaling pathways and damages critical biomolecules [85,86]. Our findings indicated that Iris-exosomes significantly reduced ROS levels in DHT-damaged HFDPCs compared with those treated with DHT alone (Figure 5). Notably, treatment with Iris-exosomes restored mitochondrial membrane potential to levels comparable to the control group (Figure 6). Furthermore, Iris-exosomes enhanced ATP activity in DHT-damaged HFDPCs, indicating a recovery of mitochondrial energy metabolism (Figure 7).

The activation of the Wnt/β-catenin signaling pathway stimulates the expression of genes involved in cell cycle progression [87]. By stabilizing β-catenin, this pathway enables its translocation into the nucleus, where it activates genes essential for hair follicle proliferation and growth [88,89]. In addition, ERK and AKT play critical roles in the Wnt/β-catenin pathway. Both proteins phosphorylate upstream components, enhancing β-catenin stability and promoting Wnt signaling [34]. Specifically, AKT inhibits GSK-3β by phosphorylating it at Ser9, thereby preventing β-catenin degradation [90]. Meanwhile, ERK amplifies β-catenin signaling, contributing to enhanced cellular responses, including hair follicle proliferation and regeneration [91]. Our study demonstrated that Iris-exosome treatment increased the phosphorylation levels of AKT, ERK, and GSK-3β, key regulators of the Wnt/β-catenin signaling pathway. This upregulation was accompanied by a significant increase in β-catenin expression, suggesting that Iris-exosomes stabilize β-catenin by preventing its degradation (Figure 8).

**Figure 10:**
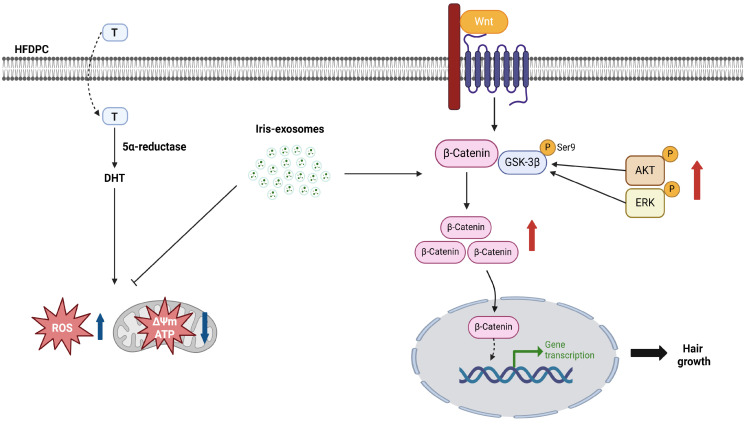
The schematic diagram illustrates how Iris-exosomes enhance hair growth. 5α-reductase converts testosterone (T) into DHT, which causes androgen-related hair loss. Iris-exosomes activate the ERK, AKT, and Wnt signaling pathways, reduce ROS levels, and restore damaged mitochondrial function, thereby contributing to the prevention and improvement of hair loss.

In conclusion, our study highlights the potential of *Iris germanica* L. rhizome-derived exosomes as a novel plant-based therapeutic approach for hair regeneration. Furthermore, our findings suggested that Iris-exosomes enhance Wnt/β-catenin signaling, which may have a positive impact on hair growth (Figure 10). These results underscore the therapeutic potential of Iris-exosomes and warrant further investigation into their applications, with clinical studies needed to validate their efficacy.

## 4. Materials and Methods

### 4.1. Preparation and Characterization of Iris-Exosomes

Iris-exosomes (ABio materials, Suwon, Republic of Korea) were obtained by the same process described in Kim et al. [92]. Dried *Iris germanica* L. rhizomes were treated with ultrahigh pressure followed by extraction using a juicer. For exosome isolation, Iris rhizome extracts were centrifuged at 10,000× *g* and 4 °C for 10 min. The collected supernatant was frozen at −80 °C for 20 h and dried for 100 h by freeze-drying under vacuum conditions. After adding distilled water to the lyophilized supernatant, mixing with an aqueous two-phase system containing 3.3% polyethylene glycol and 1.7% dextran was performed. Additional centrifugation of the mixture at 1000× *g* for 10 min at 4 °C was conducted. The supernatant was removed, and the lower-layer solution was further purified using an additional aqueous two-phase system of the same concentration. The final exosome-concentrated layer was collected after washing three times. For experiments, freeze-dried forms of collected exosomes were obtained.

### 4.2. Dynamic Light Scattering

The size distribution of Iris-exosomes was determined by dynamic light scattering (DLS), using Zetasizer Pro (Malvern, UK). To evaluate the stability of Iris-exosomes, the sizes of the exosomes were measured after 37 °C storage for 0, 24, and 48 h. Samples were prepared by diluting them with distilled water to a final volume of 1 mL and subsequently transferred to a cuvette (DTS0012, Malvern, UK). Triplicate repeat measurements were conducted at 25 °C.

### 4.3. Cryo-TEM Analysis

Grids (Lacey/Carbon 200 Mesh, Copper, EMS, Hatfield, PA, USA) were made of hydrophilic surfaces with the glow discharge system (PELCO easiGlow™, Ted Pella, Redding, CA, USA). In total, 3 μL of Iris-exosomes was added to the grid and blotted for 3 s at 100% humidity and a temperature of 4 °C. Then, the sample underwent plunge-freezing for vitrification using a Vitrobot Mark IV (Thermo Fisher Scientific, Waltham, MA, USA) in liquid ethane. The samples were analyzed by Glacios (Thermo Fisher Scientific, Waltham, MA, USA) at 200 kV.

### 4.4. Nanoparticle Tracking Analysis

The number of Iris-exosome particles was assessed using a Nanosight NS300 Nanosight LM10-HS nanoparticle characterization system (Malvern, UK). The samples were diluted in PBS to the appropriate concentration, followed by being injected into the 405 nm laser chamber using a syringe. The chamber temperature was set to 25 °C and maintained automatically. Three recordings were performed for each sample. NTA software (version 2.3) was used to measure the concentration of nanoparticles [93].

### 4.5. Protein Quantification of Iris-Exosomes

The protein concentration of the exosome preparations was determined to be 90.66 µg/mL using a Pierce™ BCA Protein Assay Kit (Thermo Fisher Scientific, Waltham, MA, USA). Absorbance was measured at 562 nm using a microplate reader (BioTek Multi-Mode Microplate Reader, Winooski, VT, USA). The particle-to-protein ratio of the exosome preparation was approximately 4.85 × 10^8^ particles per microgram of protein, indicating a relatively high purity of the sample.

### 4.6. Cell Culture

HFDPCs (Promo Cell, Heidelberg, Germany) were cultured in follicle dermal papilla cell growth medium (Promo Cell, Heidelberg, Germany) and 1% penicillin/streptomycin (Welgene Inc., Gyeongsan, Republic of Korea) at 37 °C in a 5% CO_2_ incubator.

### 4.7. Cell Viability Assay

HFDPCs were treated with Iris-exosomes (10^6^, 5 × 10^6^, 10^7^, 5 × 10^7^, and 10^8^ particles/mL). The cells were washed after 24 h of incubation. A reaction reagent from the EZ-cytox kit (DoGenBio, Seoul, Republic of Korea) was added to each well, followed by a 1 h incubation. Absorbance was measured at 450 nm for each sample.

### 4.8. Wound Healing Assay

HFDPCs were incubated in a 5% CO_2_ environment for 24 h. The center of the well was scratched using a 1 mL pipette tip. After aspirating the culture medium, the cells were treated with 1 µM DHT (Sigma-Aldrich, St. Louis, MO, USA), Iris-exosomes (10^7^ and 10^8^ particles/mL), and 1 µM MIX (Sigma-Aldrich, St. Louis, MO, USA) followed by incubation for 24 h. To achieve final concentrations of 10^7^ and 10^8^ particles/mL, 2 µL or 20 µL of a 10^10^ particles/mL stock solution were added to 2 mL of medium, respectively. Images were captured at 0, 24, and 48 h post-treatment using a Nikon light microscope (Tokyo, Japan) to assess wound closure.

### 4.9. Alkaline Phosphatase Staining Assay

Staining of alkaline phosphatase was performed using an alkaline phosphatase staining kit (Abcam, Cambridge, UK). HFDPCs were incubated for 24 h in a 5% CO_2_ incubator. Following incubation, the cells were treated with 1 µM DHT, Iris-exosomes (10^7^ and 10^8^ particles/mL), and 1 µM MIX for 24 h at 37 °C. To achieve final concentrations of 10^7^ and 10^8^ particles/mL, 2 µL or 20 µL of a 10^10^ particles/mL stock solution were added to 2 mL of medium, respectively. Following treatment, the cells were fixed using a fixing solution for 2 min. The cells were then stained with the alkaline phosphatase staining solution for 24 h, followed by washing with Dulbecco’s phosphate-buffered saline (DPBS) (Welgene Inc., Gyeongsan, Republic of Korea). Purple-stained colonies were observed and counted using a Nikon light microscope (Tokyo, Japan), and the results were compared with colorless colonies.

### 4.10. DCF-DA ROS Assay

ROS production quantification was performed using a Cellular ROS assay kit (Abcam, Cambridge, UK). HFDPCs were incubated for 24 h in a 5% CO_2_ incubator. The cells were then treated with 1 µM DHT, Iris-exosomes 10^8^ particles/mL, and 1 µM MIX for 24 h. To achieve a final concentration of 10^8^ particles/mL, 20 µL of a 10^10^ particles/mL stock solution was added to 2 mL of medium. After treatment, the cells were incubated with 10 µM 2′,7′-dichlorofluorescin diacetate (DCF-DA) for 20 min. The cells were subsequently washed with DPBS. Fluorescence was observed using a Nikon Eclipse Ti2 fluorescence live-cell imaging microscope (Tokyo, Japan).

### 4.11. Quantitative Real-Time Polymerase Chain Reaction

HFDPCs were incubated for 24 h at 37 °C in a 5% CO_2_ incubator. The cells were treated with Iris-exosomes (10^7^ and 10^8^ particles/mL), 1 µM MIX for 24 h, and 1 µM DHT for 6 h. The cells were washed twice with DPBS (Welgene, Gyeongsan, Republic of Korea) before RNA extraction. RNA was isolated using TRIzol reagent (Thermo Fisher Scientific, Waltham, MA, USA), and 2 μg of total RNA was converted into cDNA using the RevertAid First Strand cDNA synthesis kit (Thermo Fisher Scientific, Waltham, MA, USA). The assays were performed utilizing TaqMan Universal Master Mix II, with UNG, for quantitative real-time polymerase chain reaction (qRT-PCR). The reaction mixture consisted of DEPC water (6 μL), TaqMan Universal Master Fast Mix II (10 μL), cDNA (3 μL), and catalase primer (1 μL) or GAPDH primer for normalization.

### 4.12. Immunofluorescence Analysis

HFDPCs were incubated for 24 h in a 5% CO_2_ incubator. The cells were treated with Iris-exosomes (10^8^ particles/mL), 1 µM MIX for 24 h, and 1 µM DHT for 6 h. Cells were fixed with 4% paraformaldehyde for 10 min and permeabilized with 0.1% Triton X−100 for 15 min at room temperature. After blocking with 3% bovine serum albumin for 1 h, cells were incubated with a primary antibody against phosphorylated Nrf2 overnight at 4 °C. Subsequently, they were incubated with an HRP-conjugated secondary antibody for 1 h at room temperature. Finally, the nuclei were counterstained with DAPI for 15 min. Fluorescence was observed using a Nikon Eclipse Ti2 fluorescence live-cell imaging microscope (Tokyo, Japan).

### 4.13. Measurement of Mitochondrial Membrane Potential

Mitochondrial membrane potential was assessed using a JC-1 Mitochondrial Membrane Potential Assay Kit (Abcam, Cambridge, UK). HFDPCs were plated and incubated for 24 h in a 5% CO_2_ incubator. The cells were treated with 1 µM DHT, Iris-exosomes 10^8^ particles/mL, and 1 µM MIX for 24 h at 37 °C. To achieve a final concentration of 10^8^ particles/mL, 20 µL of a 10^10^ particles/mL stock solution was added to 2 mL of medium. Following treatment, the cells were stained with 5 µM JC-1 solution for 10 min. The cells were subsequently washed with DPBS. Fluorescence was observed using a Nikon Eclipse Ti2 fluorescence live-cell imaging microscope (Tokyo, Japan).

### 4.14. ATP Assay

Mitochondrial ATP levels were measured using ATP Red™ and MitoLite™ Green FM (AAT Bioquest, Pleasanton, CA, USA). HFDPCs were plated and incubated for 24 h in a 5% CO_2_ incubator. The cells were treated with 1 µM DHT, Iris-exosomes 10^8^ particles/mL, and 1 µM MIX for 24 h. To achieve a final concentration of 10^8^ particles/mL, 20 µL of a 10^10^ particles/mL stock solution was added to 2 mL of medium. Following treatment, the cells were stained with ATP Red™ working solution for 30 min. Following another wash with DPBS, the cells were stained with MitoLite™ Green FM staining solution for 30 min. The cells were subsequently washed with DPBS. Fluorescence was observed using a Nikon Eclipse Ti2 fluorescence live-cell imaging microscope (Tokyo, Japan).

### 4.15. Western Blot Analysis

HFDPCs were incubated for 24 h in a 5% CO_2_ incubator. The cells were treated with 1 µM DHT, Iris-exosomes (10^7^ and 10^8^ particles/mL), and 1 µM MIX for 24 h. To achieve final concentrations of 10^7^ and 10^8^ particles/mL, 10 µL or 100 µL of a 10^10^ particles/mL stock solution were added to 10 mL of medium, respectively. Following treatment, the cells were rinsed twice with DPBS and lysed using a RIPA lysis and extraction buffer (Thermo Fisher Scientific, Waltham, MA, USA). Protein concentrations were determined using a Pierce™ BCA Protein Assay Kits (Thermo Fisher Scientific, Waltham, MA, USA). A total of 30 µg of protein extracted from the cell lysates was prepared for analysis. The proteins were separated by sodium dodecyl sulfate–polyacrylamide gel electrophoresis (SDS-PAGE) at 120 V for 2 h and subsequently transferred overnight onto a polyvinylidene fluoride membrane (Roche, Mannheim, Germany). After blocking, the primary antibodies targeting p-AKT (4060S), AKT (9272S), p-ERK (9101S), and ERK (9102S) (Cell Signaling Technology, Beverly, CA, USA) were diluted 1:1000 in blocking solution. Primary antibodies against β-catenin (SC-59737) and p-GSK-3β (SC-373800) (Santa Cruz Biotechnology, Dallas, TX, USA) were diluted 1:500 in blocking solution. These primary antibodies were incubated overnight. The membrane was washed three times using TBS-T (Bio-Rad Inc., Hercules, CA, USA). HRP-linked antibodies (Cell Signaling Technology, Beverly, CA, USA) were applied for 1 h and then washed three times. Protein bands were detected using an ECL reagent (Cytiva, Marlborough, MA, USA), and images were acquired using the Invitrogen iBright 1500 system (Waltham, MA, USA) and analyzed with Fiji ImageJ (Win 64-bit) software, version 1.53e.

### 4.16. 3D Spheroid Formation of HFDPCs

HFDPCs were seeded and incubated for 24 h in a 5% CO_2_ incubator. Following incubation, the cells were treated with 1 µM DHT, Iris-exosomes (10^7^ and 10^8^ particles/mL), and 1 µM MIX at 37 °C in a CO_2_ incubator. To achieve final concentrations of 10^7^ and 10^8^ particles/mL, 0.2 µL or 2 µL of a 10^10F^ particles/mL stock solution were added to 200 µL of medium, respectively. Each spheroid was measured using a Nikon light microscope (Tokyo, Japan).

### 4.17. Statistical Analysis

Statistical analyses were conducted using GraphPad Prism version 8.01 (San Diego, CA, USA), with statistical significance set at *p*-value < 0.05. A Tukey’s multiple comparison test was applied after one-way ANOVA for multiple group comparisons. The results are expressed as the mean ± standard deviation (SD) based on three independent experiments.

## Data Availability

The data presented in this study are available on request from the corresponding author.
